# Digit Ratio (2D:4D) and Physical Performance in Female Olympic Athletes

**DOI:** 10.3389/fendo.2020.00292

**Published:** 2020-05-12

**Authors:** Emma Eklund, Lena Ekström, John-Olof Thörngren, Magnus Ericsson, Bo Berglund, Angelica Lindén Hirschberg

**Affiliations:** ^1^Department of Women's and Children's Health, Division of Obstetrics and Gynecology, Karolinska Institutet, Stockholm, Sweden; ^2^Department of Laboratory Medicine, Division of Clinical Pharmacology, Karolinska Institutet, Karolinska University Hospital, Stockholm, Sweden; ^3^Department of Internal Medicine, Karolinska Institutet, Karolinska University Hospital Solna, Stockholm, Sweden; ^4^Department of Gynecology and Reproductive Medicine, Karolinska University Hospital, Stockholm, Sweden

**Keywords:** 2D:4D ratio, testosterone, physical performance, androgen metabolites, female athletes

## Abstract

**Background:** The second to fourth digit ratio (2D:4D ratio) is suggested to be a negative correlate of prenatal testosterone. Little is known about the role of the 2D:4D ratio in relation to serum and urinary androgens for physical performance in female athletes. We aimed to compare the 2D:4D ratio in female Olympic athletes with sedentary controls, and to investigate the 2D:4D ratio in relation to serum and urinary androgens and physical performance in the athletes.

**Methods:** This cross-sectional study included 104 Swedish female Olympic athletes participating in power, endurance and technical sports and 117 sedentary controls. The 2D:4D ratio was calculated using direct digit measurements. Serum androgens and urinary androgen metabolites were analyzed by liquid chromatography-tandem mass spectrometry. The athletes performed standardized physical performance tests and body composition was established by dual-energy X-ray absorptiometry.

**Results:** The 2D:4D ratio was significantly lower in the athletes compared with controls although serum testosterone levels were comparable between groups and within normal reference values. The 2D:4D ratio correlated negatively with urinary levels of testosterone glucuronide and 5α- and 5βAdiol-17G, whereas there were no correlations to serum androgen levels. Furthermore, the 2D:4D ratio correlated negatively with strength tests and positively with 3,000-meter running in the athletes.

**Conclusion:** Female Olympic athletes had a lower 2D:4D ratio, possibly reflecting a higher prenatal androgen exposure, than sedentary controls. Furthermore, the 2D:4D ratio was related to urinary levels of androgen metabolites and physical performance in the athletes but not to serum androgen levels. It is suggested that the 2D:4D ratio could reflect androgen metabolism and may be of importance for sporting success in female athletes.

## Introduction

Prenatal testosterone and estrogen levels are suggested to influence the formation of the second to fourth digit ratio (2D:4D ratio), with high environmental levels of androgens during fetal life being associated with a low 2D:4D ratio (1–3). However, this concept has recently been debated (4–7). The digit ratio, proposed as a sexually dimorphic trait (2, 8, 9), is believed to be set during the first trimester of fetal development (2, 10) and does not change substantially with age (9). Furthermore, the 2D:4D ratio has been associated with Differences of Sex Development (DSD) (11, 12).

The digit ratio has also been suggested to be related to physical performance (13, 14). Previous studies have shown that male athletes have a lower 2D:4D ratio than non-athletes (15–19). However, only a few previous studies have investigated the digit ratio in female athletes in comparison to controls (15, 19–21). An association between the 2D:4D ratio and physical performance have been demonstrated in female athletes for alpine skiing (18), endurance running (22) fencing (23) and rowing (24). Similarly, positive correlations between the 2D:4D ratio and physical fitness (25), and sporting ability (26) have been demonstrated in women taking part in leisure sports. These reports are either based on rather small study groups and/or not including populations of Olympic athletes.

Androgens are considered beneficial for athletic performance by exerting positive effects on muscle tissue, erythropoiesis, immune system, and behavioral patterns, and may also contribute to a decreased risk of injuries and increased health status in athletes (27). In women, the active androgens testosterone and dihydrotestosterone (DHT) are synthesized in the ovaries and the adrenal glands, and by conversion in peripheral tissue of precursor androgens produced in the adrenal cortex, such as androstenedione (A4), dehydroepiandrosterone (DHEA), its sulfate (DHEAS) and 5-androstene-3β, 17β-diol (5-DIOL) (28). Androgens are finally mainly metabolized by uridine diphospho (UDP)-glucuronosyl transferases (UGTs) and to some extent by sulfotransferases (SULTs) and excreted in urine (29).

We have recently published data showing that female Olympic athletes have higher levels of serum androgen precursors compared to controls and that serum androgens are positively associated with physical performance in the athletes (30). These results are of relevance for the ongoing discussion regarding hyperandrogenism in female athletes (27). Adult serum androgens have also been studied in relation to the 2D:4D ratio in non-athletic populations of men and women, demonstrating inconclusive results (31, 32). No previous studies have examined the 2D:4D ratio in relation to the androgen profile in both serum and urine, as well as physical performance in female top athletes.

The aim of the present study was to investigate the 2D:4D ratio in female Olympic athletes and untrained controls, and to study the 2D:4D ratio in relation to the androgen profile in serum and urine and physical performance in the athletes.

## Materials and Methods

### Study Population

The present study included a representative population of Swedish female Olympic athletes (*n* = 104) participating in the summer or winter Olympic games, and 117 controls, for whom digit measurements were obtained (30). The controls were age- and body mass index (BMI)—matched, having a maximum of 2 h endurance and/or strength training per week and no prior participation in elite level competition. All participants were > 18 years of age. Olympic athletes were recruited in connection with pre-Olympic training camps and controls recruited via advertisement (recruitment was conducted from November 2011 to April 2015). The subjects were investigated at the Women's Health Research Unit, Karolinska University Hospital or in connection with pre-Olympic training camps. All participants filled out a general health questionnaire including training hours per week, and hormonal contraceptive use and for the athletes' information concerning sport discipline, age at training debut and age at elite level debut was obtained. Data on menstrual function was collected via questionnaire and confirmed by measurement of serum hormones for participants not using hormonal contraceptives. Blood- and urine samples were collected in a fasted, rested state, between 07.00 and 10.00 am and stored at −20°C until further analysis.

The project was approved by the Regional Ethics Committee (EPN 2011/1426-32). Informed written consent was obtained from all participants.

### 2D:4D Ratio

**Figure 1:**
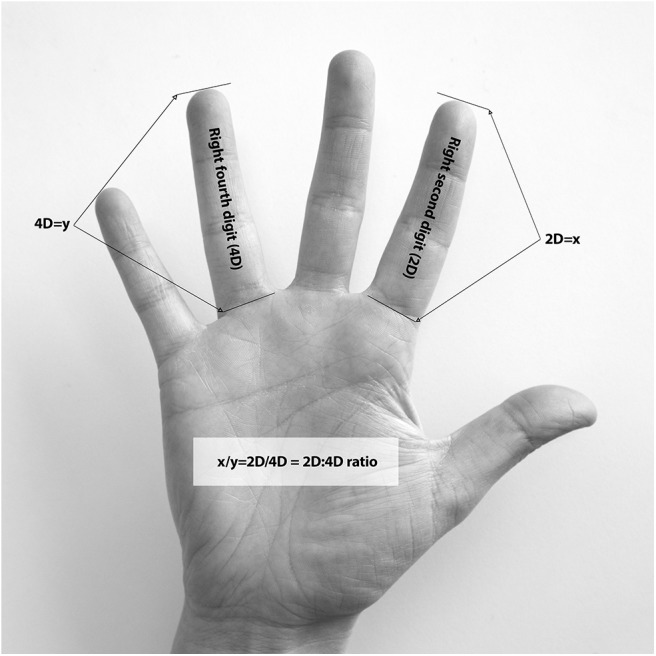
Measurement and calculation of the second to fourth digit (2D:4D) ratio.

Digit measurement expressed in millimeters (mm) was performed for digit two (2D) and digit four (4D) (Figure 1) using a Vernier digital caliper 0–150 mm (USA, Cocraft) with a precision of 0.01 mm. Digit length was directly measured from the mid-point of the proximal crease of the proximal phalanx to the distal tip of the distal phalanx for 2D and 4D (9, 15) on both left (*n* = 103 athletes, *n* = 116 controls) and right hand (*n* = 104 athletes, *n* = 117 controls). The 2D:4D ratio was calculated by dividing 2D length by 4D length. In addition, right minus left 2D:4D ratio (Dr-l), suggested as an additional negative marker for prenatal testosterone, was calculated (2). The digits were independently measured by two raters. For rater A, the intraobserver agreement was 0.90 for right hand and 0.90 for left hand. For rater B, the corresponding intraobserver agreement was 0.93 and 0.96, respectively. The inter-rater correlation was 0.87 for the right hand and 0.85 for the left hand (33).

### Body Composition

For 65 athletes and 100 controls, body composition [bone mineral density (BMD) (g/cm^2^), fat mass (%), lean mass (kg)] was established by dual-energy X-ray absorptiometry (DXA), Lunar Prodigy Advance (GE Healthcare, Madison, WI) at the Karolinska University Hospital, Solna as previously described (30).

### Physical Performance

Athletes were offered standardized physical performance tests managed by the SOC at Bosön, Stockholm, Sweden. They participated in physical performance tests measuring explosive power [countermovement jump (CMJ) (*n* = 57), squat jump (SJ) (*n* = 58)], strength (bench press (*n* = 45), chins (*n* = 49)) and 3,000 meters running (*n* = 20).

### Serum Steroid Profile

Serum levels of androgens [testosterone, DHEA, DHEAS, DHT, A4 and 17- alpha-hydroxyprogesterone (17-OHP)] and estradiol (E2) were determined by liquid chromatography-tandem mass spectrometry (LC-MS/MS) at the Endoceutics laboratory, Quebec, Canada, as previously described (34). Free androgen index was calculated (testosterone nmol/L divided by sex hormone-binding globulin (SHBG) nmol/L ^*^ 100). Follicle- stimulating hormone (FSH), luteinizing hormone (LH), anti-müllerian hormone (AMH) and SHBG were determined by electrochemiluminiscence immunoassay at the Department of Clinical Chemistry, Karolinska University Hospital as previously described (30).

### Urinary Steroid Profile

For 93 athletes, urinary samples were obtained and urinary levels of conjugated (glucuronide and sulfated) androgens [testosterone (T-G, T-S), epitestosterone (EpiT-G, EpiT-S), androsterone (ADT-G, ADT-S), etiocholanolone (Etio-G, Etio-S), DHEA-G, DHEA-S, 5α-androstane-diol (5αAdiol-3G and 5αAdiol-17G) and 5β-androstane-diol (5βAdiol- 3G and 5βAdiol-17G)] were determined by LC-MS/MS as previously described (35) at the accredited doping laboratory, Department of Laboratory Medicine, Karolinska Hospital Huddinge. Specific gravity (SG) was measured for all urine samples by a Digital Urine SG Refractometer (ATAGO UG1, Tokyo, Japan). Using a correction formula, Ccorrected = Cmeasured ^*^ ((1.020–1)/(SG−1)), each sample was corrected to a specific gravity of 1.020, adjusting for urine dilution. Limit of detection (LOD) was estimated to be below 0.4 μg/mL for all analytes. For two athletes a urine sample was not obtained and for nine athletes the amount of urine was insufficient for analysis. Testosterone:epitestosterone (T:E) ratio was calculated by dividing testosterone glucuronide (T-G) by epitestosterone glucuronide (EpiT-G).

### Statistical Analyses

Statistical analyses were performed using Statistica™ 13 software (Statsoft® Inc., Tulsa, OK, USA). Continuous data was presented as mean ± SD or as median and interquartile range (25th−75th percentile) depending on distribution. Comparison of the 2D:4D ratio and body composition between groups was performed using the student's *t*-test. The proportion of women using hormonal contraceptives and having menstrual dysfunction was calculated by Chi-Square test and type of sport by Fisher's exact test. Effect size for continuous variables was calculated using Cohen's ***d***and for categorical variables with Phi = √(Chi-2/n). Correlations were evaluated by Spearman's rank- order correlation or Pearson's correlation. *P*-values < 0.05 were considered significant.

## Results

### General Characteristics of Female Athletes and Controls

Female athletes and controls were similar regarding age and BMI (Table 1). As expected, training hours/week was significantly higher among athletes compared to controls (Table 1). Age at training debut was 9.34 ± 4.74 years and age at elite debut was 17.56 ± 3.32 years for the athletes. As previously published, hormonal contraceptive use was similar between groups, but menstrual dysfunction was significantly more common among female athletes compared to controls (30). Furthermore, the Olympic athletes demonstrated a more anabolic body composition, including higher total BMD, lower body fat percent and higher amount of lean mass compared to the controls (30) (Table 1). In addition, as previously published, the athletes had significantly lower levels of estrone and higher serum levels of the androgen precursors DHEA and 5-DIOL than controls (30). However, both groups had serum steroid levels within the normal range (30).

**Table 1:** General characteristics and body composition in Olympic athletes and controls.

**Parameter**	**Controls**	**Athletes**	**Effect size^**a**^**
*n*	117	104	
Age#	26.2 ± 5.5	25.9 ± 5.6	0.044
BMI#	22.0 ± 2.6	22.0 ± 2.0	0.008
HC use (*n* (%)#	46 (39)	40 (38)	0.009
MD (*n* (%)#	3 (4)	15 (23)**	0.28
Training (hour/week)	0.93 ± 0.85	17.93 ± 5.67***	4.32
**Digit ratio**			
2D:4D ratio right^b^	0.98 ± 0.04	0.97 ± 0.03*	0.29
2D:4D ratio left^c^	0.97 ± 0.03	0.97 ± 0.03	0.052
Dr-l	0.02 ± 0.03	0.01 ± 0.03**	0.383
**Body composition**			
N	100	65	
Total BMD (g/cm^2^)#	1.15 ± 0.07	1.25 ± 0.08***	1.315
Body fat (%)#	31.7 ± 6.6	18.4 ± 5.9***	2.108
Lean mass total (kg)#	40.4 ± 4.1	49.9 ± 5.9***	1.964

*Values presented as mean ± SD or percentage. 2D:4D ratio right, Second to Fourth digit ratio right hand; 2D:4D ratio left, Second to Fourth digit ratio left hand; BMD, Bone Mineral Density; BMI, Body Mass Index; Dr-l, right – left 2D:4D ratio; HC, Hormonal Contraceptives; MD, Menstrual dysfunction*.

# *Data previously published (30), ^a^ Cohen's d (continuous variables) or Phi (categorical variables), ^b^ data available for calculation for 104 athletes and 117 controls, ^c^ data available for calculation for 103 athletes and 116 controls*.

*p < 0.05, **p < 0.01, ***p < 0.001.

### 2D:4D Ratio in Relation to Serum and Urinary Androgen Levels in Athletes and Controls

**Figure 2:**
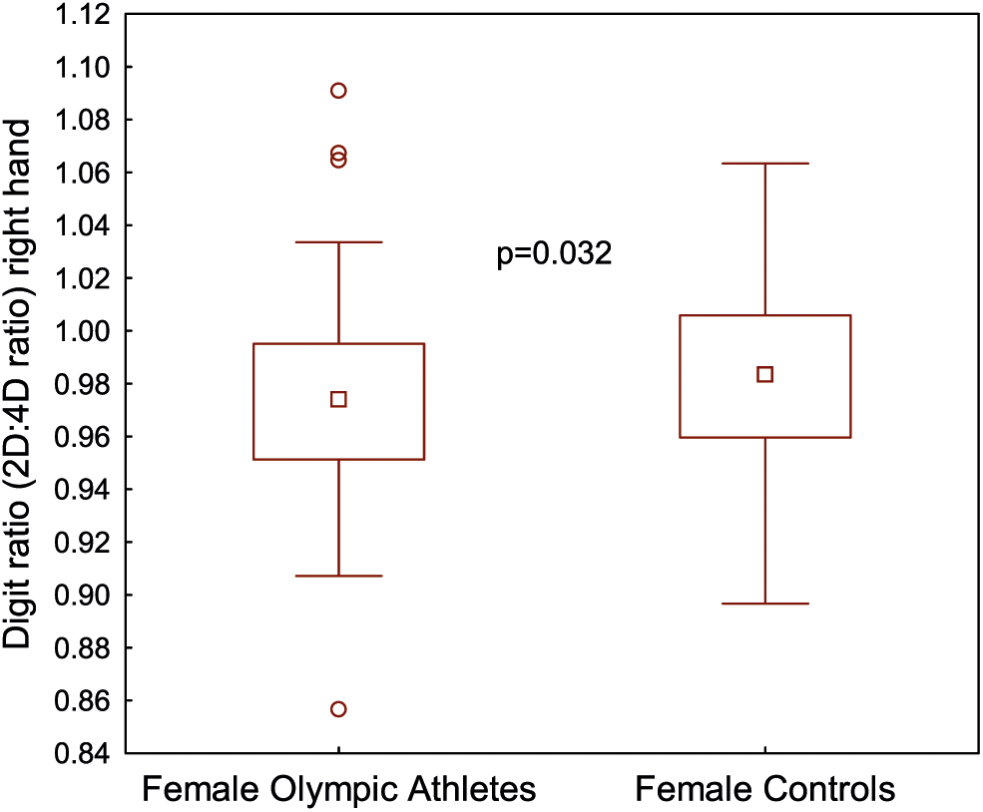
Digit ratio (2D:4D ratio) right hand expressed in millimeters (mm) for female Olympic athletes (*n* = 104) and female controls (*n* = 117).

**Figure 3:**
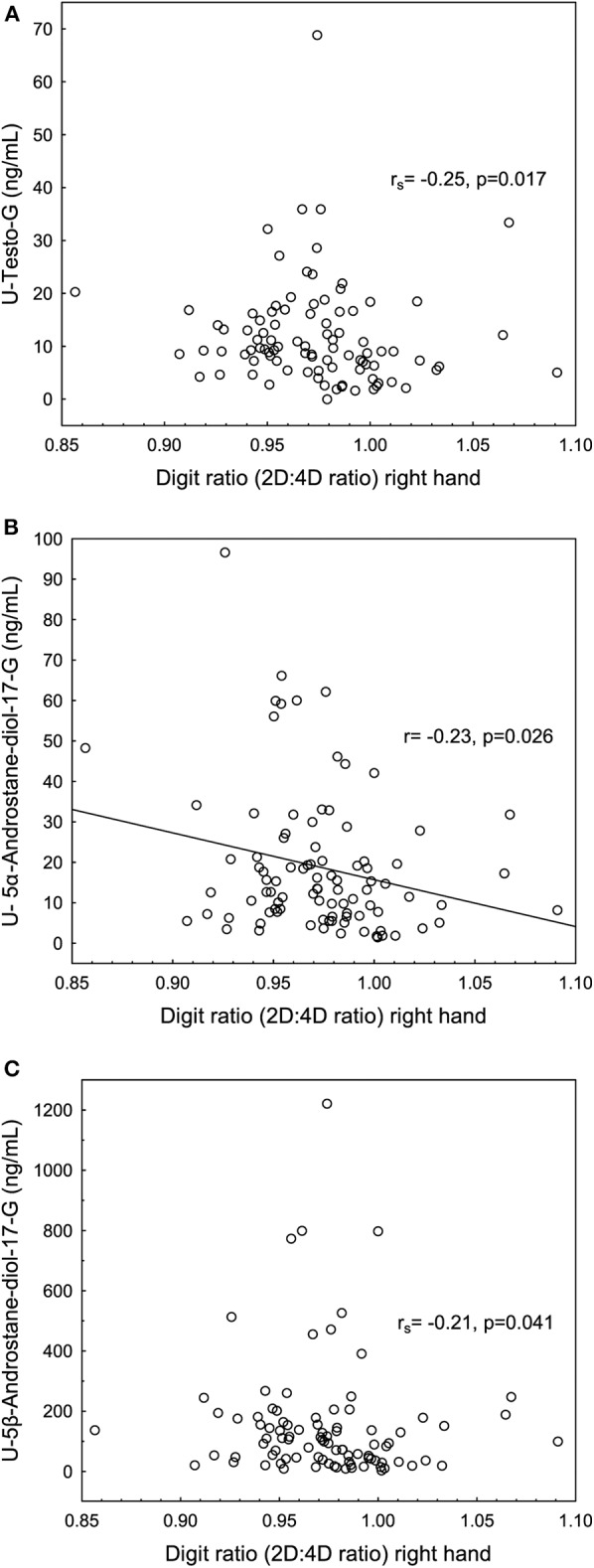
Significant negative correlations between the second to fourth digit ratio (2D:4D ratio) right hand expressed in millimeters (mm) and **(A)** urine testosterone glucuronide (U-Testo-G) (ng/mL), **(B)** U−5α- Androstane-diol-17G (5aAdiol- 17G) and **(C)** U-5β- Androstane-diol-17G (5βAdiol-17G) in female Olympic athletes.

The 2D:4D ratio right hand was significantly lower in the female Olympic athletes than the controls (*p* < 0.05) (Table 1, Figure 2). Furthermore, the athletes demonstrated a significantly lower Dr-l compared to the controls (Table 1). No significant correlations were found between serum androgen levels and the 2D:4D ratio in the controls or the female Olympic athletes. However, in the athlete group, there were significant negative correlations between the 2D:4D ratio right hand and the urinary steroid metabolites T-G, 5αAdiol-17G and 5βAdiol-17G, see Figures 3A–C. However, there were no corresponding correlations for Dr-l.

### 2D:4D Ratio, Urinary Androgens and Physical Performance in Athletes

**Figure 4:**
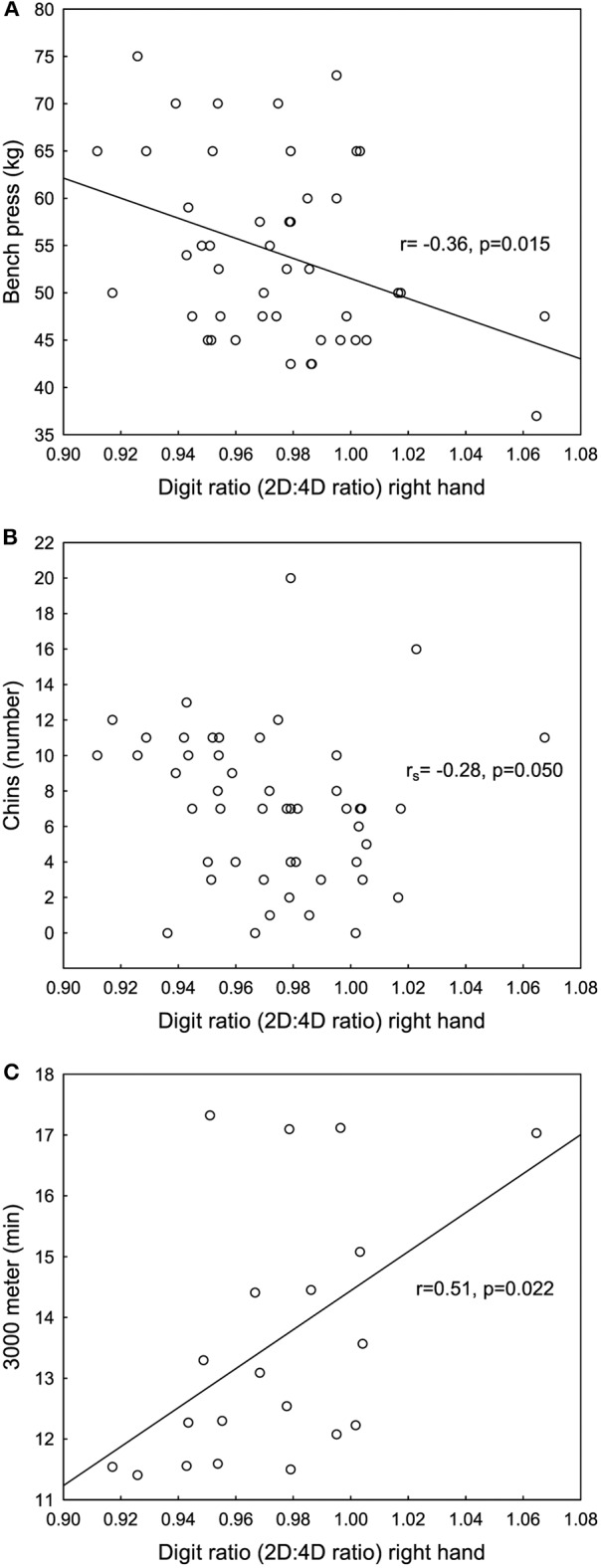
Significant negative correlations between the second to fourth digit ratio (2D:4D ratio) right hand and **(A)** bench press and **(B)** chins and a significant positive correlation between the 2D:4D ratio right hand and **(C)** 3,000 meters running measured in minutes for female Olympic athletes.

Significant negative correlations were found between the 2D:4D ratio right hand and bench press and chins, see Figures 4A,B and a significant positive correlation between the 2D:4D ratio right hand and 3,000 meters running performance see Figure 4C. No significant correlations were found between Dr-l and physical performance.

In turn, 5αAdiol-3G and 5βAdiol-17G correlated positively to chins (r_s_ = 0.33, *p* < 0.05 and r_s_ = 0.37, *p* < 0.05, respectively) and Etio-G correlated negatively to running time 3,000 m (r_s_ = −0.51, *p* < 0.05).

## Discussion

To our knowledge, this is the first study investigating the 2D:4D ratio in relation to both the serum and urinary androgen profile and physical performance in female top athletes. We found a lower 2D:4D ratio right hand, suggesting a higher prenatal androgen exposure, in female Olympic athletes than untrained controls. In addition, we found negative correlations between the 2D:4D ratio and the urinary steroid profile, and both these variables correlated significantly with physical performance including strength tests and middle-distance running in the female Olympic athletes.

Few previous investigations (15, 19–21) have reported data on the 2D:4D ratio in female athletes compared with controls. These studies have been performed in female athletes at national level (20), varsity athletes (non- elite athletes) (15), college tennis players (21) and youth handball players (19) demonstrating a significantly lower digit ratio for the athletes compared to controls. However, our study is the first including a large number of female Olympic athletes and untrained controls. In agreement with most previous studies, we found significant differences in the 2D:4D ratio only for the right hand (15, 19, 21). It has been demonstrated that the right hand shows a greater sex difference than the left hand leading to the suggestion that the right hand is more representative of prenatal androgen influence (8).

The relation between prenatal androgen exposure and the 2D:4D ratio is supported by an association between a low 2D:4D and high levels of fetal testosterone relative to estradiol in amniotic fluid in humans (1). Furthermore, male human fetuses have higher androgen levels in amniotic fluid (36) and significantly lower 2D:4D ratio than female fetuses (10). However, a relationship between the 2D:4D ratio and adult sex hormone levels have not been confirmed (31, 32). In agreement, we found no correlation between serum androgen levels and the 2D:4D ratio. More recent studies suggest that the digit ratio is not correlated to resting serum androgen levels, but could be associated with androgen levels in response to physical training (2, 37). However, in our study data was collected in a resting state and not in close connection to training or competition.

On the other hand, we found for the first time, significant negative correlations between the 2D:4D ratio and several urinary androgen metabolites (T-G, 5αAdiol-17G, 5βAdiol-17G). Our findings could reflect a possible difference in androgen phase II metabolism depending on 2D:4D ratio among female athletes. As opposed to the circulatory androgens, the urinary androgen concentrations are dependent on the expression and activity of phase II enzymes i.e., UGTs and SULTs. T-G, 5βAdiol-17G and 5αAdiol-17Gs are all conjugated at the 17β- OH-position preferable by UGT2B17, whereas T-G and 5αAdiol-17G may also be inactivated by UGT2B15, UGT2A1, and UGT1A4 (38, 39). The expression and activity of UGT2B17 and UGT2B15 are known to be higher in men than in women (40, 41). It is possible that polymorphisms in UGTs and other androgen metabolizing enzymes, as well as other factors that determine the expression and activity of UGTs, may be associated with the androgen load of the fetus in the first trimester. Several fetal UGTs are expressed already in the first trimester (42). Further studies are warranted to establish any putative link between phase II metabolism and the 2D:4D ratio.

In support of a role of prenatal androgen exposure for physical performance, we found significant correlations between the 2D:4D ratio and athletic performance tests in the Olympic athletes. Previous studies on 2D:4D ratio and physical performance have focused on male athletes (16, 17, 22), whereas there is more limited research in female athletes. The few previous studies in female athletes have demonstrated significant correlations between a lower 2D:4D ratio and faster rowing times (24), faster skiing times (18), better endurance running performance (22) and better national fencing rank (23). In our study of top-level female athletes, the 2D:4D ratio was significantly related to both strength tests and 3,000 m running, a test mainly representative of aerobic capacity.

There are several possible underlying mechanisms for the association between the 2D:4D ratio and athletic performance. We and others have previously demonstrated associations between adult serum androgen levels and muscle mass and strength in female athletes (30, 43, 44). However, we found no correlations between serum androgen levels and the 2D:4D ratio. In contrast, there were correlations between the 2D:4D ratio and urinary steroid metabolites, which in turn correlated to physical performance tests. As urinary androgen levels are dependent on androgen metabolizing enzymes, these urinary metabolites are more representative for the androgen metabolism than circulating androgens. Furthermore, the androgen metabolism is dependent on genetic variations. We hypothesize that genetic variation of the androgen metabolism and thereby androgen activity, could influence both the development of the 2D:4D ratio during fetal life and the predisposition for physical performance.

In conclusion, we found a lower 2D:4D ratio related to urinary androgen levels and physical performance in female Olympic athletes. The association between the digit ratio and urinary androgen levels, but not serum androgen levels, may indicate that the 2D:4D ratio reflects variations in androgen metabolism rather than absolute circulating androgen levels. A low digit ratio was associated with increased aerobic and strength performance in the athletes. Although the link between the 2D:4D ratio and physical performance, is still not fully clarified, our results suggest that prenatal androgen exposure, in addition to the adult androgen levels, may be of importance for athletic capacity in female Olympic athletes.

## Data Availability Statement

All datasets generated for this study are included in the article/supplementary material.

## Ethics Statement

The studies involving human participants were reviewed and approved by the Regional Ethics Committee (EPN 2011/1426-32). The patients/participants provided their written informed consent to participate in this study.

## Author Contributions

EE, BB, and AH were involved in the concept/design of the study, acquisition of data, and data analysis. J-OT and ME performed the quantification of urinary androgens. EE, BB, LE, J-OT, ME, and AH were involved in the manuscript preparation, critical revision of the article and approval of the article. All authors listed met the conditions required for full authorship.

## Conflict of Interest

BB is the medical director for the Swedish Olympic Committee (SOC) and AH is medical adviser to the SOC, the International Association of Athletic Federation (IAAF) and the International Olympic Committee (IOC). The results of the study are presented clearly, honestly, and without fabrication, falsification, or inappropriate data manipulation. The remaining authors declare that the research was conducted in the absence of any commercial or financial relationships that could be construed as a potential conflict of interest.
